# Efficient Co-Replication of Defective Novirhabdovirus

**DOI:** 10.3390/v8030069

**Published:** 2016-03-04

**Authors:** Ronan N. Rouxel, Emilie Mérour, Stéphane Biacchesi, Michel Brémont

**Affiliations:** VIM, INRA, Université Paris-Saclay, Jouy-en-Josas 78350, France; ronan.rouxel@jouy.inra.fr (R.N.R.); emilie.merour@jouy.inra.fr (E.M.); stephane.biacchesi@jouy.inra.fr (S.B.)

**Keywords:** Novirhabdovirus, defective particles, fluorescent proteins

## Abstract

We have generated defective Viral Hemorrhagic Septicemia Viruses (VHSV) which express either the green fluorescent protein (GFP) or a far-red fluorescent protein (mKate) by replacing the genes encoding the nucleoprotein N or the polymerase-associated P protein. To recover viable defective viruses, rVHSV-ΔN-Red and rVHSV-ΔP-Green, fish cells were co-transfected with both deleted cDNA VHSV genomes, together with plasmids expressing N, P and L of the RNA-dependent RNA polymerase. After one passage of the transfected cell supernatant, red and green cell foci were observed. Viral titer reached 10^7^ PFU/mL after three passages. Infected cells were always red and green with the very rare event of single red or green cell foci appearing. To clarify our understanding of how such defective viruses could be so efficiently propagated, we investigated whether (i) a recombination event between both defective genomes had occurred, (ii) whether both genomes were co-encapsidated in a single viral particle, and (iii) whether both defective viruses were always replicated together through a complementation phenomenon or even as conglomerate. To address these hypotheses, genome and viral particles have been fully characterized and, thus, allowing us to conclude that rVHSV-ΔN-Red and rVHSV-ΔP-Green are independent viral particles which could propagate only by simultaneously infecting the same cells.

## 1. Introduction

The presence of defective interfering (DI) viral particles during replication in infected cells is a well-known phenomenon in the virology field [1]. Genomes of DI particles are deleted during the replication and can further replicate into the infected cells only in the presence of co-infection with complete wild-type helper virus. DI genomes are encapsidated into neo-particles and propagate by interfering with wild-type virus replication [2]. Generally, the appearance of DI is the result of cell infection at high multiplicity of infection [3]. During passages in the cell culture of a mixture containing wild-type virus and DI particles, the viral titers progressively decrease. One plausible explanation for this observation is that the kinetics of replication of DI, due to the smaller size of the genome, is faster than for wild-type virus. DI and helper virus exist for most of the virus families including Novirhabdovirus, although they are understudied [4,5]. Apart from DI, viral RNA genome may in some cases be rearranged. For the positive strand RNA virus, that rearrangement is very frequent, mainly because, during viral replication, RNA genome is naked in the cytoplasm and the RNA polymerase may jump from one replicative genome to another one [6,7]. In contrast, for negative strand RNA virus, recombination event has never been described, with a single exception for Respiratory Syncytial Virus for which a co-infection with two replication-competent viruses, knock-out for NS1/NS2 and G genes, resulted in the generation of a virus with a rearranged genome [8]. A number of papers have described recombination events for another negative-strand RNA virus, Newcastle Disease Virus [9,10,11,12,13,14,15,16], however, these descriptions are controversial since in several examples these recombination events are only artificial and due to errors in the deposited Genbank sequences [17]. Finally, as it has been described mainly for Measles Virus, in some cases, a single virus particle may encapsidate more than one genome and stably propagate the different genomes [18,19]. This might be due to the pleomorphic structure of Paramyxoviruses. In contrast, viruses belonging to the Rhabdovirus family like Vesicular Stomatitis Virus or rabies virus and Novirhabdovirus present a rigid bullet shape containing a single RNA molecule [20]. Novirhabdovirus like the Viral Hemorrhagic Septicemia Virus (VHSV) are fish rhabdovirus infecting a large spectrum of fish species, mostly trout, thus replicating at low temperatures. The genome of Novirhabdovirus is about 11 Kbases consisting of a negative sense single-stranded RNA molecule which encodes five structural proteins, the nucleoprotein N, a polymerase-associated P protein, the matrix M protein, a unique G glycoprotein and the large L RNA-dependent RNA polymerase. In addition, located between the G and L genes, Novirhabdovirus genomes present an additional short gene encoding a non-structural NV protein which has been shown to be involved in the viral pathogenicity [21,22]. As demonstrated by reverse-genetics, only N, P and L proteins are needed for formation of transcriptionally active rhabdovirus nucleocapsids [23]. In the current study, we have generated by reverse genetics two recombinant replication-defective Novirhabdoviruses, deleted each for a gene essential for the replication encoding either the N or the P protein. Thus, VHSV-derived cDNA genomes have been engineered such as the N or P genes have been exchanged with reporter genes encoding the green fluorescent protein (GFP) or the red monomeric mKate protein, respectively [24,25]. The aim of this study was to investigate if a complementation phenomenon allowing efficient replication of both defective VHSV might exist.

## 2. Materials and Methods

### 2.1. Cells and Virus

Recombinant wild-type like VHSV designated rVHSV [26] and rVHSV-GFP (see below) were propagated in monolayer cultures of *Epithelioma papulosum cyprinid* (EPC) cells at 14 °C as previously described [27]. Virus titers were determined by plaque assay on EPC cells under an agarose overlay (0.35% in Glasgow’s modified Eagle’s medium-25 mM HEPES medium supplemented with 2% fetal bovine serum and 2 mM L-glutamine). At 5–7 days post-infection, cell monolayers were fixed with 10% formol and stained with crystal violet. Recombinant vaccinia virus expressing the T7 RNA polymerase, vTF7-3 [28], was kindly provided by B. Moss (National Institutes of Health, Bethesda, MD, USA).

### 2.2. Recovery of rVHSV-GFP

A plasmid construct pVHSV-dtTomato containing VHSV-derived genomic cDNA with an additional dtTomato gene (Clontech) between N and P VHSV genes [26] was digested with *SpeI/SnaBI* restriction enzymes to remove and to replace the dtTomato gene with the GFP gene derived from the pMAX-GFP expression plasmid (Amaxa). The rVHSV-GFP was rescued following transfection of pVHSV-GFP together with pT7-N, pT7-P and pT7-L into vTF7-3-infected EPC cells as previously described [26]. All the restriction enzymes are from Thermo Fisher Scientific (Villebon-sur-Yvette, France).

### 2.3. Plasmid Constructs Encoding Defective VHSV-Derived cDNA Genomes

A pVHSV plasmid construct containing the full length VHSV-derived genomic cDNA [26] was used as a DNA matrix to amplify by PCR two DNA fragments *SacII/PsiI* (containing the N gene) and *PsiI/MfeI* (containing the P gene); using pairs of primers SACPSIF/SACPSIR and SPEMFEF/SPEMFER, respectively (Table 1).

Both DNA fragments were cloned into a pJet1.2 (Thermo Fisher Scientific). *SpeI* and *SnaBI* restriction enzyme sites were introduced at the start and stop codons, respectively, of the N and P genes by site-directed mutagenesis using QuikChange^®^ Site-Directed Mutagenesis Kit (Agilent, Les Ullis, France) and specific primers SPEMUTN and SNABIMUTN (for N) and SPEMUTP and SNABIMUTP (for P) (Table 1). Each of the mutagenized DNA fragments were digested with *SpeI/SnaBI* restriction enzymes to remove N and P genes and exchanged them by mKate and GFP genes, respectively. *SacII/PsiI* and *PsiI/MfeI* DNA fragments containing either mKate or GFP genes were reintroduced back into two separate pVHSV constructs leading to pVHSV-ΔN-Red and pVHSV-ΔP-Green plasmids, respectively.

### 2.4. Recovery of Defective rVHSV Expressing mKate and GFP Genes

pVHSV-ΔN-Red and pVHSV-ΔP-Green plasmid constructs (1 μg each) were transfected into vTF7-infected EPC cells together with pT7-N, pT7-P and pT7-L as previously described [26]. One week later, cell supernatant was used to infect fresh EPC cells. Three days post-infection cells were observed under UV-light microscope (Leica, Nanterre, France).

### 2.5. Plaque Purification

Recombinant VHSV-ΔN-Red and VHSV-ΔP-Green were titered under an agarose overlay as described above. At 6 days post-infection, plaques were observed under UV-light microscope. Plaques appearing exclusively red or green were recovered by aspiration with micropipette and resuspended in 200 µL 2% complete medium before EPC cells infection. Complete medium (2%) was added after infection and cells were incubated at 15 °C until apparition of cytopathic effect.

### 2.6. RT-PCR on Plaque-Purified Viruses

Genomic viral RNA was extracted from infected-cell supernatant after total cytopathic effect using the QIAamp Viral RNA Purification Kit (QIAGEN, Courtaboeuf, France) according to the manufacturer’s instructions. Viral RNA was then reverse-transcribed using Reverse Transcriptase IV (Thermo Fisher Scientific) with VHSCDNA primer and then amplified by PCR using primers VHSPCRF and VHSPCRR (Table 1). PCR products of roughly 1800 and 2300 nucleotides (nt) for rVHSV-ΔN-Red and for rVHSV-ΔP-Green RNA genomes, respectively, were purified with PCR Purification Kit (QIAGEN) following the manufacturer’s instructions and cloned into pJet1.2 (Thermo Fisher scientific). Positive clones were selected and subjected to sequencing with PJETFOR, PJETREV and VHSNF primers (Table 1).

### 2.7. Purification of rVHSV and rVHSV-ΔN-Red + rVHSV-ΔP-Green Particles on Sucrose Gradient

All the viruses were mass produced in EPC cells and supernatants of the infected cells were clarified by low speed centrifugation (4000 rpm 15 min). Supernatants (35 mL) were first concentrated by ultracentrifugation in a SW28 Beckman rotor (Beckman Coulter, Villepinte, France) at 25,000 rpm for 90 min at 4 °C, resuspended in cell culture medium without fetal serum and then loaded on a 25% sucrose cushion in TEN buffer 1× (10 mM Tris-HCl pH 7.5, 150 mM NaCl, 1 mM EDTA pH 8). After ultracentrifugation at 36,000 rpm in a SW41 Beckman rotor (Beckman Coulter, Villepinte, France) for 4 h at 4 °C, viral pellets were resuspended in 100 μL of TEN, loaded onto a 15%–45% discontinuous sucrose gradient, and ultracentrifuged overnight at 25,000 rpm in a Beckman SW55 rotor (Beckman Coulter) at 4 °C. Unique bands of purified viral particles were collected, diluted in TEN and pelleted by ultracentrifugation in a SW41 Beckman rotor at 25,000 rpm for 90 min at 4 °C. Final pellets were resuspended in TEN (60 μL) and stored at −80 °C until further use.

### 2.8. Virus Preparation and Electron Microscopy Observations

As above, rVHSV, rVHSV-ΔN-Red + rVHSV-ΔP-Green and rVHSV-GFP were mass produced in EPC cells, clarified by low centrifugation at 4000 RPM for 15 min and were ultracentrifugated at 25,000 rpm in a SW28 Beckman rotor (Beckman Coulter) for 90 min at 4 °C. Pellets were carefully resuspended in TEN buffer in appropriated volume to obtain 100 fold concentration rate. Four microliters of purified viruses were added to a Formvar-coated EM grid (300 meshs), incubated 5 min and then contrasted with 1% aluminium molybdate pH: 8 for 20–30 s. All steps were performed at room temperature. The grids were observed using a transmission electron microscope HITACHI HT7700 (Elexience-France, Verrières-le-buisson, France) operated at 80 kV. Microphotographies were acquired with a charge-coupled device CCD camera 8 million pixels and analyses were done with Hitachi HT7700-associated program (Advanced Microscopy Techniques Corp, Woburn, MA, USA). Viral particles were measured with for length and width (nm) and data were represented by scatter plots (Graphpad Prism 5) with statistical Tukey’s Multiple Comparison Test analysis.

## 3. Results

### 3.1. Recovery of Defective Recombinant Viral Hemorrhagic Septicemia Virus

**Figure 1:**
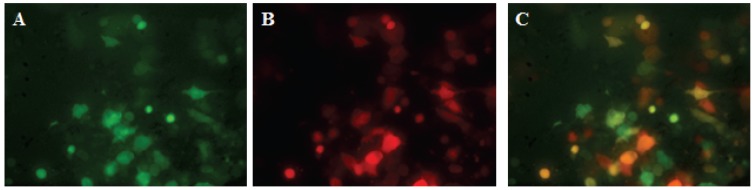
Observation under UV-light microscope of defective rVHSV-infected cells. EPC cells were infected with the supernatant P0 from vTF7-3-infected cells transfected with pVHSV-ΔN-Red and pVHSV-ΔP-Green plasmid together with pT7-N, pT7-P and pT7-L. Infected cells were observed under UV-light microscope at wavelength of 509 nm for green fluorescence (**A**) and 633 nm for far-red fluorescence (**B**). Overlapping picture of green and red infected-cell foci (**C**).

Following EPC cell infection with vTF7 and transfection of the two plasmid-constructs pVHSV-ΔN-Red (mKate) and pVHSV-ΔP-Green (GFP) together with the helper expression plasmids pT7-N, pT7-P and pT7-L, the cell supernatant (P0) was used to infect fresh EPC cells. Three days later, when cells were observed under UV-light microscope, a large number of red and green cell foci could be seen, the vast majority of them being yellow after overlapping of Red and Green fluorescence, although the intensity of each signal was variable from one infected cell to the other (Figure 1). Comparison of the panels A and B clearly emphasizes that infected cells are always both green and red.

When supernatant of infected cells (P1) was further passaged several times, a similar observation was made, except for the appearance of some cell foci exclusively Red or Green (see below). The rescue of replication-defective viruses rVHSV-ΔN-Red + rVHSV-ΔP-Green was a very efficient process since for three out of four independent transfection assays the recovery of recombinant viruses was successful.

### 3.2. Genome Characterization of Virus from Red and Green Infected-Cells

**Figure 2:**
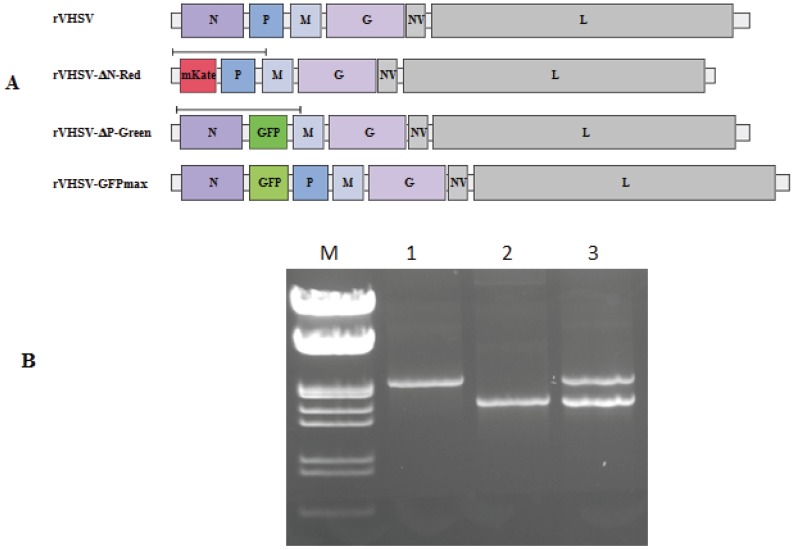
Schematic representation of the various recombinant VHSV genomes and RT-PCR products analysis. A schematic representation of rVHSV, rVHSV-ΔN-Red (mKate), rVHSV-ΔP-Green (GFP) or rVHSV-GFP genomes is shown. Part of the genomes containing mKate or GFP genes was amplified through RT-PCR with specific primers (Table 1). The black line above genomes indicates the parts of the genomes amplified (**A**). Agarose gel analysis of the PCR or RT-PCR products amplified from either the plasmid constructs pVHSV-ΔP-Green (1) and pVHSV-ΔN-Red (2) or from supernatant of yellow infected-cell foci (3), respectively (**B**). M: DNA molecular weight marker (ThermoFisher scientific).

Supernatant of rVHSV-ΔN-Red- and rVHSV-ΔP-Green-infected cells was collected and viral genomic RNA was extracted and served to amplify using specific primers by RT-PCR part of the genomes containing either mKate or GFP genes (Figure 2A).

As a positive control, the initial pVHSV-ΔN-Red and pVHSV-ΔP-Green plasmid constructs were used as DNA template and amplified by PCR with the same primers as above. Analysis on agarose gel of the PCR and RT-PCR products indicated that the expected size for the PCR products of 1761 nt and 2280 nt for rVHSV-ΔN-Red and rVHSV-ΔP-Green, respectively, have been amplified (Figure 2B). That evidenced that a mix of the initial defective RNA genomes were still present in the viral RNA extracted from the infected-cell supernatant and also that no apparent genome rearrangement has occurred.

### 3.3. Characterization of Red and Green Cell Foci

**Figure 3:**
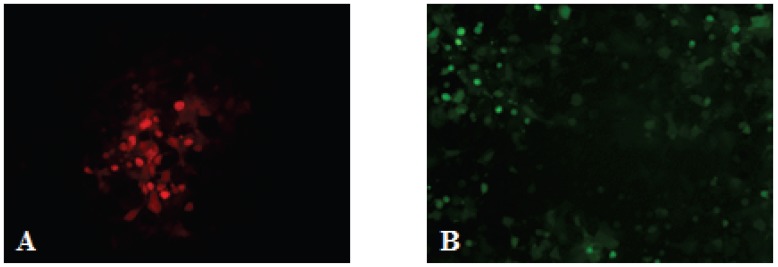
Fluorescence of Red or Green infected-cell foci on same field. At passage five, EPC cells were infected with rVHSV-ΔN-Red + rVHSV-ΔP-Green (m.o.i = 0.0001). Three days later, the same field of infected cells was examined for Red and Green fluorescence. Some of the infected cell foci appeared exclusively Red (**A**) or Green (**B**). A counting, mean of two independent experiments, of the monochromatic infected cell foci indicated an estimate of 9.6% of the total foci.

As indicated above, after three to five passages, some infected cell foci were exclusively Red or Green (Figure 3).

These cell foci were plaque purified under agarose, visualized under UV-light microscope and isolated by picking them and propagated in 24-well plaques. That confirmed that indeed some infected cells after observations under UV-light microscope were exclusively Red or Green. Supernatant of these infected cell cultures were collected and viral genomic RNA was extracted and used to amplify by RT-PCR, as above, part of the genomes containing the mKate or GFP genes. Analysis of agarose gel of the PCR products showed as above that two products of 1761 nt and 2280 nt have been amplified, indicating that the two distinct viral genomes (ΔN and ΔP) were present. The nucleotide sequencing of these purified RT-PCR products indicated that several mutations leading to non-conservative amino acid changes appeared in the mKate and GFP genes (Table 2).

These mutations may explain that although being co-infected with the two rVHSV-ΔN-Red + rVHSV-ΔP-Green, some cells appeared exclusively Green or Red. To ascertain that these mutations leaded up to deleterious effect on the reporter protein fluorescence, both mutated mKate and GFP genes were cloned into a CMV-driven eukaryotic expression vector (ThermoFisher Scientific) and transfected into fish cells. Two days later when transfected-cells where examined under UV-light microscope, no fluorescence could be observed in contrast to the cells transfected with non-mutated reporter genes. That demonstrated that the mutations observed in both reporter genes completely abolished the fluorescence of the expressed proteins.

### 3.4. Viral Particles Content and Morphology

**Figure 4:**
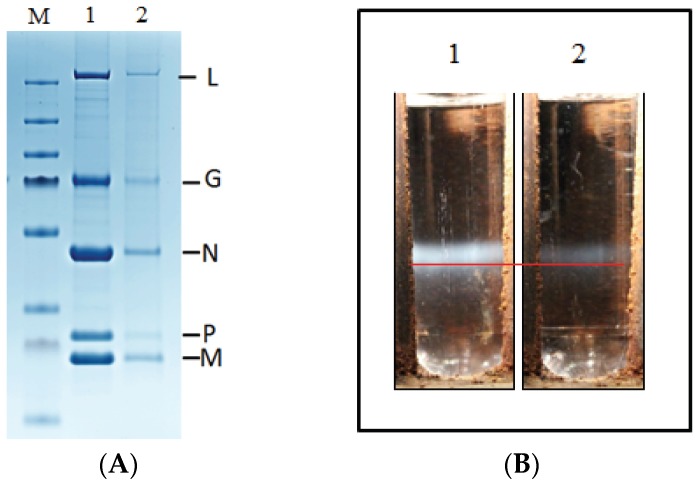
SDS-PAGE analysis and purification on sucrose gradient of recombinant VHSV. Semi-purified rVHSV and rVHSV-ΔN-Red + rVHSV-ΔP-Green on 25% sucrose cushion were analyzed through protein separation onto a 4%–12% SDS-PAGE (**A**). After Coomassie blue staining, a similar viral protein pattern for rVHSV (panel 1) and VHSV-ΔN-Red + rVHSV-ΔP-Green (panel 2) could be observed. On the right part, name of VHSV proteins (L, G, N, P and M) are indicated; M: molecular weight marker (ThermoFisher scientific). Discontinuous 15%–45% sucrose gradients were loaded with semi-purified viral stocks (**B**). After ultracentrifugation, unique bands, sedimenting at the same position in the gradient (red line), were visible for rVHSV (1) and rVHSV-ΔN-Red + rVHSV-ΔP-Green (2).

To further characterize the defective rVHSV, supernatant of infected cells was loaded on sucrose cushion as described in Materials and Methods. Protein contents of the resuspended virus pellets were analyzed through separation on a 4%–12% SDS-PAGE. Following Coomassie-blue staining, a similar viral protein pattern could be observed between rVHSV and the mix of rVHSV-ΔN-Red + rVHSV-ΔP-Green (Figure 4A).

**Figure 5:**
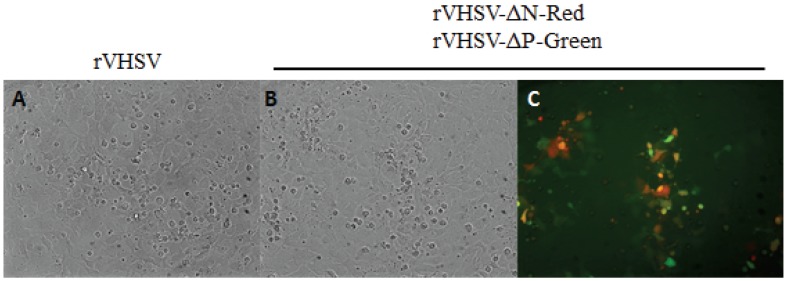
Infection of EPC cells with sucrose gradient-purified recombinant viruses. EPC cells in 24-well plates were infected (m.o.i. = 1) with rVHSV or rVHSV-ΔN-Red + rVHSV-ΔP-Green purified on sucrose gradient (see Figure 4B). Infected cells were observed two days post-infection either under phase-contrast (rVHSV and rVHSV-ΔN-Red + rVHSV-ΔP-Green, **A** and **B**, respectively) or UV-light microscope (rVHSV-ΔN-Red + rVHSV-ΔP-Green, **C**). Overlapping of Red and Green fluorescence is presented showing that majority of the infected cells were yellow (Red + Green).

To compare the density of the viral particles rVHSV-ΔN-Red + rVHSV-ΔP-Green to rVHSV, aliquots of semi-purified virus on sucrose cushion were loaded on a discontinuous sucrose gradient. Figure 4B shows that although less material is present for rVHSV-ΔN-Red + rVHSV-ΔP-Green compared to rVHSV, those particles sedimented at the same position in the gradient, indicating that density of rVHSV-ΔN-Red + rVHSV-ΔP-Green and rVHSV was equal. As above, analysis on SDS-PAGE of the viruses contained in the sucrose gradient bands revealed a similar pattern of viral proteins between rVHSV and mixture of rVHSV-ΔN-Red + rVHSV-ΔP-Green. In addition, we showed that sucrose gradient-purified rVHSV-ΔN-Red + rVHSV-ΔP-Green were still infectious when used to infect EPC cells (Figure 5).

**Figure 6:**
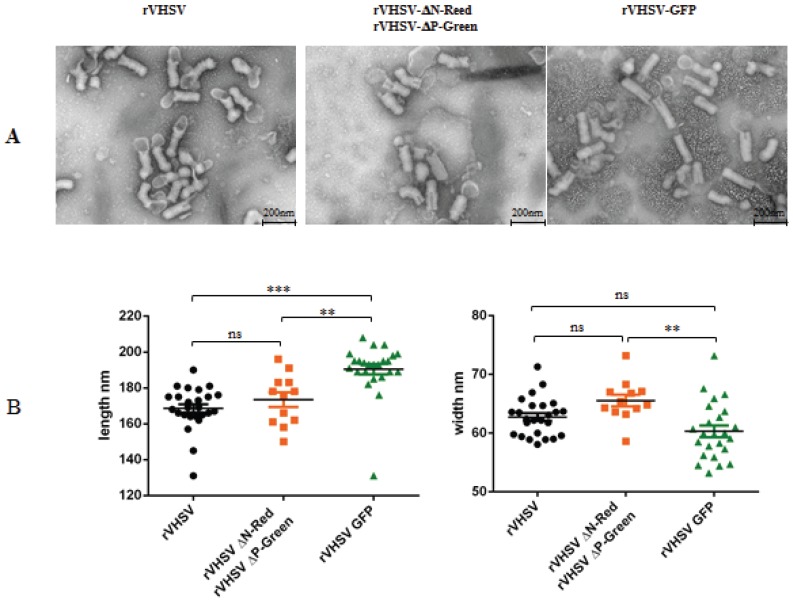
Electron microscopy observations of various recombinant VHSV. Recombinant rVHSV, rVHSV-GFP, rVHSV-ΔN-Red + rVHSV-ΔP-Green were semi-purified onto a 25% sucrose-cushion as described in Materials and Methods. Aliquots (5 μL) of the various recombinant viruses were deposited on Formvar-coated EM grid (300 meshs). Following staining with 1% aluminium molybdate, grids were observed with HITACHI HT7700 electron microscope at 25,000× (Bar = 200 nm) (**A**). Recombinant rVHSV and rVHSV-ΔN-Red + rVHSV-ΔP-Green are morphologically indistinguishable while rVHSV-GFP appeared larger in size. Individual recombinant viral particles were measured in length and in width (**B**). Data were represented by scatter plots (Graphpad Prism 5) with statistical Tukey’s Multiple Comparison Test analysis. Groups that are not significantly different from each other are noted ns (*p* > 0.05), whereas those that are significantly different are noted ** (*p* < 0.01) or *** (*p* < 0.001).

Semi-purified viruses were analyzed through electron microscopy observation. rVHSV-ΔN-Red + rVHSV-ΔP-Green were compared to the rVHSV but also to a non-defective rVHSV expressing the GFP (rVHSV-GFP) from an additional expression cassette in the viral genome (see Materials and Methods). The size of the genome of the rVHSV-GFP is increased of 750 nt compared to rVHSV, from 11,165 to 11,915 nucleotides. For rhabdovirus, the size in length of the particle increases proportionally to the size of the genome [29,30]. Figure 6A shows electron microscopy pictures of rVHSV, rVHSV-ΔN-Red + rVHSV-ΔP-Green and rVHSV-GFP. While rVHSV and rVHSV-ΔN-Red + rVHSV-ΔP-Green appear similar in size, the rVHSV-GFP seems to be larger, reflecting the larger size of the genome.

To indeed confirm this observation, individual viral particles were measured in length and in width. As shown in Figure 6B, rVHSV and rVHSV-ΔN-Red + rVHSV-ΔP-Green are strictly similar in size while rVHSV-GFP is significantly larger. That observation reinforced the idea that individual rVHSV-ΔN-Red and rVHSV-ΔP-Green particles contain a single RNA genome having the expected size in length.

## 4. Discussion

In the current study, we have shown that under particular conditions, defective Novirhabdovirus could efficiently propagate together in cell culture. For successful replication, those defective Novirhabdovirus need to be complemented for the lacking proteins, like in the current study the nucleoprotein N and the polymerase-associated P protein. To investigate if that was indeed feasible, we have generated defective recombinant VHSV expressing reporter genes, mKate a far red-fluorescent protein and GFP a green fluorescent protein easy to observe under UV-light microscope. Previous attempts to rescue defective rVHSV using stable clone cell lines expressing viral N or P proteins always failed. As a defective virus cannot by itself replicate, helper virus is needed. Thus, to generate helper virus, we reasoned that if we could provide in a single cell, defective viral genomes deleted from the N or the P genes, the defective genomes will complement each other. To achieve that, two VHSV-derived cDNA genomes were constructed—pVHSV-ΔN-Red and pVHSV-ΔP-Green—in which the N and P genes are replaced by mKate and GFP genes, respectively. Thanks to the reverse genetics system previously established in our laboratory [26] when the two plasmid constructs were transfected into fish EPC cells, yellow overlapping red and green cell foci could be visualized under UV-light microscope. This observation, potentially indicated, that recombinant defective rVHSV-ΔN-Red + rVHSV-ΔP-Green have been generated and were able to replicate and to express the reporter genes. Titer of these dual recombinant viruses was around 10^7^ PFU/mL (two log lower than the rVHSV). More interestingly, these recombinant defective viruses could be propagated and passaged in cell culture up to seven times, without a decrease in viral titers and infected-cells were still mainly yellow (Red + Green) with the exceptions of some infected cell foci exclusively Red or Green. To explain how this very efficient complementation phenomenon leading to the propagation of two defective recombinant virus expressing a reporter gene could be possible, we speculated three main hypotheses: (i) a recombination event leading to a rearranged viral genome in a single particle has occurred; (ii) two defective genomes encapsidated in a single viral particle; (iii) two distinct recombinant viruses (ΔN and ΔP) always co-infect and replicate together into the same cells. To address these questions, a series of assays aiming to genetically and morphologically characterize the defective recombinant VHSV expressing the Red and Green fluorescent proteins. While the recombination is a well-known and frequent phenomenon during replication of positive-sense RNA virus, it is extremely rare and even non-existent for *Mononegavirales*. A single study on Respiratory Syncytial Virus (RSV) described the appearance of rearranged viral RNA genomes following experimental cell infection with two-defective but replication-competent recombinant RSV [8]. It was a very rare event since only in one out of six co-infection assays a rearranged genome was generated. Thus, to investigate whether a recombination event might explain the successful recovery of rVHSVs able to co-express Red and Green fluorescent proteins simultaneously in the same cell, viral RNA genomes were extracted from infected-cell supernatant and characterized. The successful amplification through RT-PCR with primers localized at the beginning of the viral genome and in the M gene leading to two PCR products of the expected sizes strongly suggested that no recombination event during viral replication has occurred. That has also been confirmed by the nucleotide sequencing of each of the PCR product reflecting that the initial defective genomes’ organization was maintained during passages of rVHSV-ΔN-Red + rVHSV-ΔP-Green in cell culture. At that step, it became clear that the permanent co-expression of Red and Green fluorescent proteins during the virus passages in cell culture was not the result of a recombination phenomenon between both defective viral genomes. Another possible explanation for the effective co-existence of both rVHSV-ΔN-Red and rVHSV-ΔP-Green could be that instead of having two distinct viruses each containing a defective genome, particles containing two genomes are produced. That has been observed, for example, for Measles virus in which mutated and wild type genomes are present in the same viral particle [18,19]. To solve this question, two studies have been conducted aiming to characterize the viral particles. If two genomes (Red and Green) are co-encapsidated in a single particle, those particles should sediment at a different position in a sucrose gradient compared to particles containing a single genome. The observation that rVHSV and rVHSV-ΔN-Red + rVHSV-ΔP-Green sedimented similarly at the same position in the gradient, reinforced the hypothesis that each of the rVHSV-ΔN-Red and rVHSV-ΔP-Green particles contains a single genome. Also, this observation excluded the possible explanation that these viruses replicated while being associated and forming conglomerates. Anyway, the hypothesis of conglomerates as infectious entities has been invalidated by infecting cells with viral supernatants before and after sonication. No significant decrease of the viral titer was observed after sonication. Viruses extracted from the purified bands from the sucrose gradient were shown to be as infectious as non-purified viral supernatant, confirming that extracted band corresponded to rVHSV-ΔN-Red + rVHSV-ΔP-Green. To try to get more evidence for that “single genome” hypothesis, rVHSV-ΔN-Red + rVHSV-ΔP-Green particles were visualized by electron microscopy, allowing also to measure those viral particles. Morphologic appearance was strictly similar between rVHSV, VHSV-ΔN-Red + rVHSV-ΔP-Green and the size of the various particles identical with the exception of rVHSV-GFP. The size of rVHSV-GFP is larger in terms of length as its genome was increased in size by the addition of the GFP expression cassette. Together, these observations lead to the conclusion that the two defective recombinant rVHSV-ΔN-Red + rVHSV-ΔP-Green are co-replicating together through co-infection of the same cell. During the course of this study, we observed the appearance of infected cell foci which were either exclusively Red or Green. It was demonstrated that both rVHSV-ΔN-Red and rVHSV-ΔP-Green still co-replicated but expression of either Red or Green fluorescent proteins was abolished due to the introduction of deleterious mutations. In conclusion, in the current study, we showed for the first time, to our knowledge, that two defective *Novirhabdovirus* can very efficiently co-infect and co-replicate in the same cell and produce progenies at high titer. It will be of interest in future studies to use those recombinant viruses to infect their natural host, rainbow trout, and to follow whether these viruses are still pathogenic and whether they continue to co-replicate in the animal.

## 5. Conclusions

In the current work, we clearly demonstrated for the first time, that two defective negative strand RNA viruses can co-replicate and produce progenies at high titer. This phenomenon is independent from recombination or co-encapsidation events. If ever these recombinant defective viruses are shown to be attenuated in fish, this approach could represent an attractive alternative for the development of live-attenuated vaccine.

## Figures and Tables

**Table 1:** Primer sequences used in the study.

Primer Name	Primer Sequence (5’to 3’)
VHSCDNA	GTATCATAAAAGATGATGAGTTATGTTACAAGGG
VHSPCRF	GTTGAACACAGAGTCATATCTCATAATCG
VHSPCRR	GGTGGAGACACGGTCCTCATCATTGGACGTGAGG
PJETFOR	CGACTCACTATAGGGAGAGCGGC
PJETREV	AAGAACATCGATTTTCCATGGCAG
VHSNF	GATGACGACTACCCCGAGGACTCTGAC
SACPSIF	CTCCACCGCGGTAATACGACTCACTATAGG
SACPSIR	GCTTTGATCAAAGAGAAATTCTTATAATCGTGCCG
PSIMFEF	CGGCACGATTATAAGAATTTCTCTTTG
PSIMFER	GGCCTGCCACAATTGCCTTGACCACC
SPEMUTN	CGTTGAACAAAAGAACTCAGTACTAGTATGGAAGGAGGAATTCGTGCAGCG
SNABIMUTN	CGACTACCCCGAGGACTCTGACTAATACGTACTCCCGTCTCATAACCAACATAG
SPEMUTP	GACAAACACTGAGATACTAGTATGGCTGATATTGAGATGAGCGAGTCC
SNABIMUTP	CGATCAAGGCGGAGCTGGACAAGCTAGAGTAGTACGTACACAACGCATCACAC

**Table 2:** Amino acid changes observed in fluorescent proteins in Red and Green plaque purified viruses.

**Red Plaque-Purified Virus**	**rVHSV-ΔP-Green Clone**	F60L/Y211H (GFP)
	**rVHSV-ΔN-Red Clone**	-
**Green Plaque-Purified Virus**	**rVHSV-ΔP-Green Clone**	-
	**rVHSV-ΔN-Red Clone**	L116P/V120A/F127L/L175S/Y179H/Y195H/Y231H (mKate)
